# Electron cryomicroscopy observation of acyl carrier protein translocation in type I fungal fatty acid synthase

**DOI:** 10.1038/s41598-019-49261-3

**Published:** 2019-09-10

**Authors:** Jennifer W. Lou, Kali R. Iyer, S. M. Naimul Hasan, Leah E. Cowen, Mohammad T. Mazhab-Jafari

**Affiliations:** 1 Department of Medical Biophysics, University of Toronto, Princess Margaret Cancer Research Institute, Toronto, Ontario, Canada; 2 Department of Molecular Genetics, University of Toronto, Toronto, Ontario, Canada

**Keywords:** Pathogens, Cryoelectron microscopy

## Abstract

During fatty acid biosynthesis, acyl carrier proteins (ACPs) from type I fungal fatty acid synthase (FAS) shuttle substrates and intermediates within a reaction chamber that hosts multiple spatially-fixed catalytic centers. A major challenge in understanding the mechanism of ACP-mediated substrate shuttling is experimental observation of its transient interaction landscape within the reaction chamber. Here, we have shown that ACP spatial distribution is sensitive to the presence of substrates in a catalytically inhibited state, which enables high-resolution investigation of the ACP-dependent conformational transitions within the enoyl reductase (ER) reaction site. In two fungal FASs with distinct ACP localization, the shuttling domain is targeted to the ketoacyl-synthase (KS) domain and away from other catalytic centers, such as acetyl-transferase (AT) and ER domains by steric blockage of the KS active site followed by addition of substrates. These studies strongly suggest that acylation of phosphopantetheine arm of ACP may be an integral part of the substrate shuttling mechanism in type I fungal FAS.

## Introduction

Type I fungal FAS forms a barrel-shaped complex composed of two reaction chambers. In *Saccharomyces cerevisiae*, the *FAS1* and *FAS2* genes produce a 230 kDa β- and a 220 kDa α-chain, respectively, that assembles into a heterododecamer of α_6_β_6_^1,2^. Six β-chains form the walls of the barrel while a central wheel, made by the six α-chains, bisects the barrel into two chemically identical reaction chambers. Each chamber is formed by the central α-wheel and three β-chains around a C3 axis of symmetry. The two chambers are related by C2 symmetry, making the complex D3 symmetric (Fig. S1A). Therefore, each chamber has three complete sets of catalytic domains including three acyl-carrier protein (ACP) domains. ACP in type I fungal FAS is an 18 kDa eight helical domain composed of two four helical subdomains. One subdomain is found in type I metazoan and type II bacterial FAS ACP and herein referred to as canonical lobe. The additional four helical subdomain (herein referred to as structural lobe) is found in type I fungal and bacterial FAS. In the atomic resolution crystal structures of *S*. *cerevisiae* FAS, ACP is seen at the KS-binding site with both lobes of the domain contributing to the binding interface^1,2^. ACP interacts twice with the KS domain during each catalytic cycle, unlike other catalytic sites where the mobile domain only interacts once^3^ (Fig. S1A). Therefore, it is speculated that ACP interaction with other reaction sites is more transient. The canonical lobe is post-translationally modified with a phosphopantetheine moiety catalyzed by phosphopantetheinyl transferase (PPT) domain^4^. This reaction creates holo-ACP, which can covalently bind substrates and reaction intermediates allowing the fungal FAS to carry out the multi-step synthesis of palmitoyl-coenzyme A^3^. Except for the PPT domain, all catalytic centers face the interior of the chamber. Substrates are shuttled between the static reaction centers by the mobile ACP domain flexibly tethered at its N and C termini. A challenge in biophysical study of type I fungal FAS is experimental observation of the interaction landscape of the mobile ACP within the reaction chambers. In near-atomic resolution electron cryomicroscopy (cryoEM) maps of type I fungal and atomic-resolution cryoEM maps of type I bacterial FAS, ACP density is heterogenous as it samples multiple locations within the reaction chamber^5–7^. Therefore methods that can modulate localization of ACP within the reaction chambers of fungal FAS, may improve ACP visualization in experimental cryoEM or X-ray crystallography density maps. Here, we have experimentally probed for the ability to redistribute ACP, by stalling catalysis at the KS site in two type I fungal FASs.

## Results and Discussion

### Probing ACP location within the reaction chambers of *S*. *cerevisiae* and *C*. *albicans*

We used cryoEM to reconstruct *ab initio* ACP densities inside the reaction chambers of endogenous fungal FASs from *S*. *cerevisiae* and the opportunistic pathogen *Candida albicans* in the Apo and KS-stalled state, at 12 Å resolution, allowing localization of densities corresponding to this mobile domain (Fig. S1A–D). The *ab initio* ACP densities were generated using an ACP-less initial cryoEM density map that was generated from the ~3 Å resolution atomic model of *S*. *cerevisiae* FAS^2^ with ACP atoms deleted and low-pass filtered to 30 Å. For simplicity, we call these maps ACP-*ab initio* (AAI) maps. The AAI maps were scaled relative to each other for comparison of ACP densities between Apo and KS-stalled reconstructions for each fungal species and are shown at identical resolution range (*i*.*e*. 30–12 Å) and threshold, unless otherwise stated. The AAI maps were then refined to high resolution (Figs S2E, S2, S4, and Table S1) to probe i) ability to resolve ACP helices and its phosphopantetheine moiety and ii) for ACP-mediated conformational transitions as discussed below.

**Figure 1:**
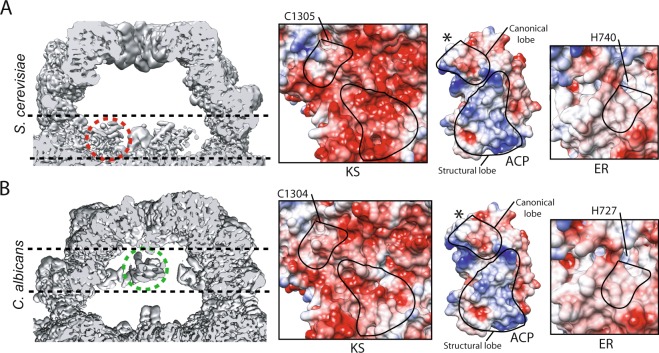
Different ACP localization within (**A**) *S*. *cerevisiae* and (**B**) *C*. *albicans* FAS in the Apo state. One ACP in each reaction chamber is highlighted with black dashed lines and red (*S*. *cerevisiae*) and green (*C*. *albicans*) circles. Coulombic surface coloring using Chimera^32^ for the interaction interfaces for KS- ACP- and ER-domains are shown. The interacting surface area for the portions of ACP’s structural and canonical lobes are encircled with continuous lines. The catalytic residues for KS and ER are highlighted and *represents the position of the phosphopantetheine arm of ACP.

In the Apo state, ACP density is strongest in proximity of the KS domain in *S*. *cerevisiae* FAS (Figs 1A and S2D,E) and allows for a complete tracing of its backbone atoms in the high resolution cryoEM map (Fig. S5A). In the Apo state of *C*. *albicans* (62 and 69% identity for β- and α-chains, relative to their respective *S*. *cerevisiae* chains), the ACP density is strongest in proximity of the ER domain, similarly allowing for complete tracing of the backbone atoms of this mobile domain at an alternate location (Figs 1B and S2D,E).

Surface electrostatics have been previously speculated to be a steering force for ACP-mediated substrate shuttling based on molecular dynamic simulations^8^. Examining the surface electrostatics of ACP- KS- and ER-domains shows a strong surface charge complementarity between the structural lobe of ACP with the KS domain in *S*. *cerevisiae* (Fig. 1A). However, the negative surface charge on KS domain is weakened in *C*. *albicans* due to alteration of some of the acidic residues that form the interface with the structural lobe of ACP (Figs 1B and S6A). A cryoEM map of a thermophilic fungal (*i*.*e*. *Chaetomium thermophilum*) type I FAS also observed ACP in proximity of the ER domain, albeit at a global resolution of 4.7 Å, which allowed for domain docking of ACP and assignment of partial density to the phosphopantetheine arm^7^. Interestingly, this pattern of weakened negative surface charge is predicted to be preserved in *C*. *thermophilum* FAS based on sequence alignment (Fig. S6A). Weaker charge complementarity can partly explain why in this pathogenic fungal species, ACP is not primarily localized at the KS in the Apo state. There are no significant alterations in the surface electrostatics of the ACP binding site in the ER domain between the *S*. *cerevisiae* and *C*. *albicans* (Fig. 1) and residues lining the ACP binding site on the ER domain are mostly conserved between the two species studied here and the *C*. *thermophilum* (Fig. S6B). Interestingly, unlike the KS-binding site where both canonical and structural lobes of ACP contribute to the surface area of the binding interface, the interface of ACP at the ER mainly involves the canonical lobe (Fig. S5B). This observation can explain the worse local resolution seen for the ACP of *C*. *albicans* localized at the ER compared to that in *S*. *cerevisiae* at the KS domain in the Apo states (Fig. S2E). It is possible that the transient interactions of ACP that are mainly driven by its phosphopantetheine-containing canonical lobe are more prone to chemical and structural alterations.

**Figure 2:**
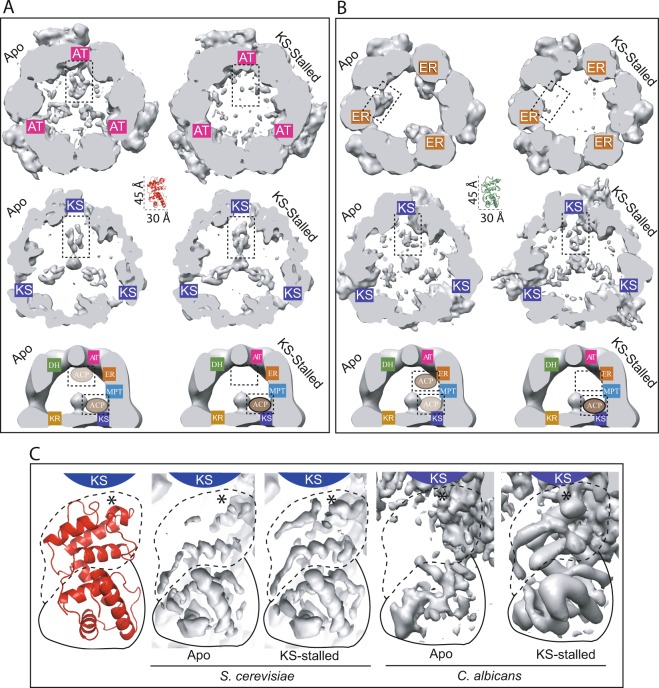
ACP distribution and translocation in *S*. *cerevisiae* and *C*. *albicans* FAS. First two row slices through the reaction chamber near (**A**) AT- and KS-domains for *S*. *cerevisiae*, and (**B**) ER- and KS-domains for *C*. *albicans* in the Apo and KS-stalled states. See Fig. S2D for position of slices. Slices for each catalytic centre from *Ab initio* maps are shown at identical resolution range and threshold in panels (**A**,**B**). Model of ACP shown to scale at the center of panels A and B in red and green for *S*. *cerevisiae* and *C*. *albicans*, respectively. Schematic representation of observed ACP translocation is shown at the bottom of each panel. (**C**) High-resolution refined maps of FAS showing improved high-resolution features of the structural (solid line) and canonical (dashed line) lobes of ACP in proximity of the KS catalytic cavity in the KS-stalled state for each fungal species. Atomic model of ACP with the same orientation as the maps is shown to the left in red. *Represent the position of the phosphopantetheine arm. The position of the KS catalytic cavity is labeled schematically in cyan.

In both fungal FASs, weaker densities can also be seen proximal to other catalytic centers in the Apo state. In *S*. *cerevisiae* FAS, second strongest ACP density is observed at lower resolution and less stringent threshold of the AAI map that shows ACP near the apical region of the reaction chamber and facing the AT catalytic site (Figs 2A and S2D). In the Apo state of *C*. *albicans* FAS, the second strongest density is in proximity of the KS domain that is only observable at lower resolution and threshold of the AAI cryoEM density map (Figs 2B and S2D). These ACP densities do not refine to high-resolution to characterize and model their respective binding interfaces in the Apo states of the two fungal FASs (Fig. S2E), most likely due to a lower occupancy and less stable interaction compared to that of ACP with the KS and ER domains in *S*. *cerevisiae* and *C*. *albicans*, respectively.

### Redistribution of ACP within the reaction chambers of *S*. *cerevisiae* and *C*. *albicans*

In type II bacterial FAS, where ACP is only composed of the canonical lobe, substrate loading state can modulate the affinity profile of a panel of ACP mutants toward the KS protein and active-site specific cross-linkers have proven to be valuable tools to trap transiently formed ACP complexes with other catalytic proteins such as ER and dehydratase (DH)^9–12^. We therefore hypothesized that the loading-state of ACP may modulate its interaction with the catalytic centers in the context of fully assembled type I fungal FAS and allow for redistribution of ACP toward a specific reaction site. Both endogenously purified enzymes are catalytically active (*i*.*e*. contain holo-ACP) and are sensitive to cerulenin, a covalent KS-inhibitor, as judged by monitoring NADPH oxidation at 340 nm (Fig. S1B). In the crystal structures of cerulenin-inhibited *S*. *cerevisiae* FAS, partial density could be seen for the inhibitor, which allowed model building for a portion of the compound^13,14^. In both our activity assays and cryoEM samples, the FAS enzymes were pre-incubated with cerulenin prior to addition of substrates acetyl- and malonyl-CoA and NADPH to ensure KS site was inhibited prior to the start of the reaction (*i*.*e*. KS-stalled state). In our high-resolution cryoEM maps, we can see appearance of additional densities immediately above the catalytic cysteine of the KS-domain in the KS-stalled state suggesting covalent linkage between cerulenin and the KS catalytic residue (Fig. S4C), which confirms our activity assay demonstrating inhibition of catalysis at the ketoacyl synthesis step (Fig. S1).

Stalling catalysis at the KS domain drastically alters the ACP densities inside the reaction chamber of both fungal FASs, as judged by improved ACP density near KS domain and weakened ACP density by the AT- and ER-domains in *S*. *cerevisiae* and *C*. *albicans* FASs, respectively (Fig. 2A,B). In both FASs in the Apo state, the structural lobe of ACP has stronger and better resolved densities compared to the canonical lobe in the KS-binding site, as observed in the high-resolution refined maps (Fig. 2C), suggesting a more stable interaction between the former and the KS-domain in the absence of substrates. In both species, the major improvement in cryoEM density of ACP at the stalled KS domain is observed at its canonical lobe (Fig. 2C). These observations suggest a bimodal interaction between ACP and the KS domain, one mediated by the ACP structural lobe and the other by its canonical lobe, that may be modulated independently by the state of the ACP- and KS- catalytic sites. In the KS-stalled state, ACP is presumably acylated with acetyl or malonyl as it can not transfer the starting (*i*.*e*. acetyl loaded by AT domain) and elongating (*i*.*e*. malonyl loaded by MPT domain) substrates to the KS from its phosphopantetheine arm. However, in our cryoEM maps of *S*. *cerevisiae* where ACP density is better resolved in proximity of the KS domain relative to *C*. *albicans*, density for the phosphopantetheine arm at the KS-catalytic cavity can only be seen until the phosphate group (Fig. S7). This is likely due to conformational heterogeneity of the phosphopantetheine arm combined with ACP’s transient interaction with the KS. Therefore, we can not experimentally observe a complete density of the acylated phosphopantetheine and can only infer its acylated state based on the catalytic competency of the enzyme and its inhibition with cerulenin (Figs S1B and S4C). The observation that stalling catalysis at one site (*i*.*e*. KS) can affect ACP localization at other sites (*i*.*e*. AT and ER) in the presence of substrates within the context of the fully assembled reaction chamber strongly suggests that substrate loading state of the mobile domain may be a contributing factor in ACP-mediated substrate shuttling in type I fungal FAS.

### ACP-dependent conformational changes in the enoyl reductase catalytic site

**Figure 3:**
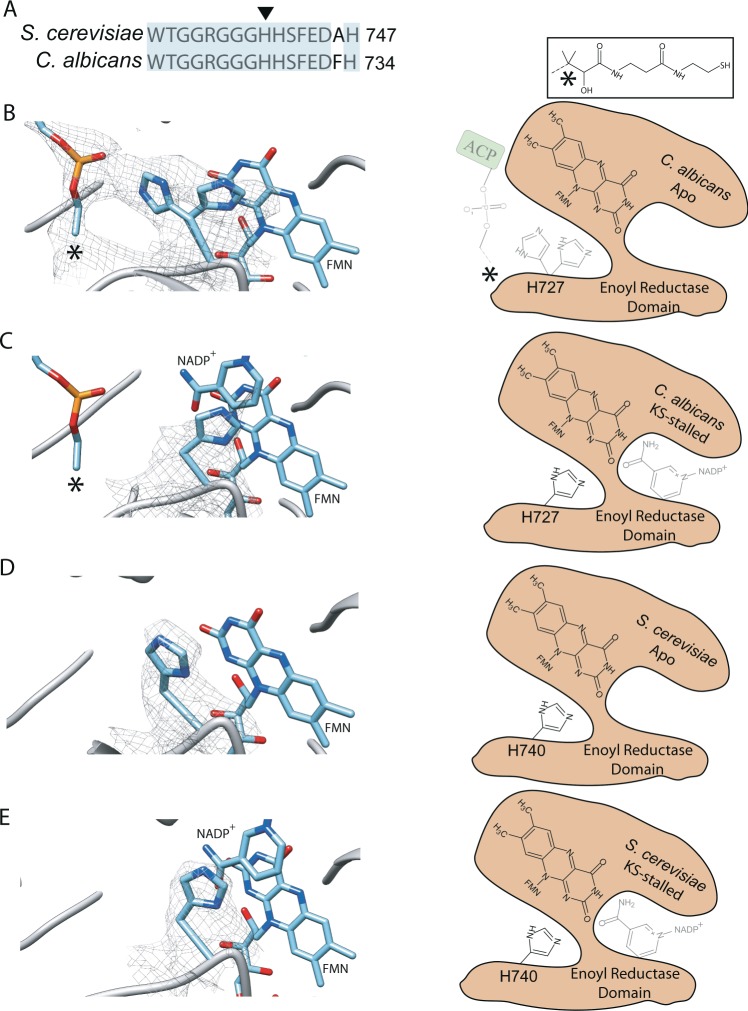
Conformational rearrangement of catalytic histidine of the ER-domain upon ACP-binding. (**A**) Sequence alignment of the ER catalytic loop between the two fugal FAS with the conserved region highlighted in light cyan. Catalytic histidine shown with arrow head. cryoEM densities are shown within 3.5 Å of the of catalytic histidine and the atoms of the phosphopantetheine arm as shown in the (**B**) Apo- and (**C**) KS-stalled states of *C*. *albicans*. (**D**,**E**) Are cryoEM densities of the catalytic histidine, shown within 3.5 Å in the Apo- and KS-stalled state of *S*. *cerevisiae* FAS, respectively. For clarity, densities of FMN and NADPH are not shown. The schematic diagrams of the ER catalytic site are shown to the right for each panel. *Represents the unmodeled portion of the phosphopantetheine prosthetic group. Transparent schematics represent partial occupancy.

With the ability to observe ACP at the ER and redirect this mobile domain to the KS, we focused on conformational differences within the ER catalytic region of *C*. *albicans* FAS. This site within *C*. *albicans* enzyme represented the most pronounced alteration in ACP localization upon stalling catalysis at the KS-domain (Fig. S2D,E). The ER domain shows the highest sequence and structural divergence between type I human and fungal FAS and is an ideal drug target for infectious diseases including *Candida* infections in immune compromised patients. Unlike human FAS^15^, the fungal ER domain uses a flavin mononucleotide (FMN) co-factor for the conversion of enoyl-ACP to saturated acyl-ACP. The FMN is then replenished by NADPH that can bind a pocket accessible from outside the reaction chamber^16^. The histidine residue adjacent to FMN is proposed to catalyze proton transfer based on homology to FMN-dependent bacterial protein FabK^17,18^. The loop hosting the catalytic histidine is highly conserved between *S*. *cerevisiae* and *C*. *albicans* (Fig. 3A).

Examining the ER active site in *C*. *albicans* Apo state where ACP is partially bound, we can clearly see the density of the phosphopantetheine arm until the quaternary carbon atom of the moiety (Fig. 3B). Interestingly, the catalytic histidine can be modeled with two side chain orientations, one facing FMN and the other facing the phosphate group of the phosphopantetheine arm. When ACP is shifted away from the ER domain in the KS-stalled state, the catalytic histidine is mainly in one conformation facing FMN (Fig. 3C). Weak density can be seen for the NADPH in proximity of FMN in the KS-stalled state, indicating partial/transient binding that does not clash with either of the histidine side chain conformations seen here (Fig. S8A). Interestingly, in the *S*. *cerevisiae* Apo and KS-stalled states, where no ACP density can be seen by the ER domain, the catalytic histidine side chain is in a single conformation facing FMN (Fig. 3D,E). Weak NADPH density can also be seen in proximity of FMN in the KS-stalled *S*. *cerevisiae* FAS (Fig. S8B). During catalysis, the enoyl group of the elongating fatty acid chain should come in proximity of the catalytic histidine to enable the reduction reaction. Based on these structures, the catalytic histidine in the ER domain of type I fungal FAS may sample alternative conformations to form a proton transfer bridge between FMN and the enoyl functional group of the elongating fatty acid chain when ACP is bound.

## Conclusion

Structural knowledge on the interaction landscape of ACP within type I FAS and understanding the mechanism of ACP-mediated substrate shuttling are highly sought after in antibiotic development^19–21^ and biofuel production efforts^22–24^. This study demonstrates that ACP interaction with a catalytic center can be modulated by stalling catalysis at another reaction site, suggesting that the loading state of ACP’s phosphopantetheine arm may have at least a partial role in determination of ACP localization within the reaction chambers of type I fungal FAS. Future structural studies should systematically investigate ACP distribution upon inhibition of each reaction site in FAS (Fig. S1A) using site-specific inhibitors or point mutations in recombinantly expressed enzymes.

## Materials and Methods

### Yeast strains and protein Purifications

The genomic DNA of haploid protease deficient *S*. *cerevisiae* strain BJ2168 (MATa leu2 trp1 ura3–52 prb1-1122 pep4-3 prc1-407 gal2) was modified by homologous recombination method^25,26^ to encode for a 3×FLAG tag at the C terminus of *FAS1* gene. To construct the modified yeast strain (JWL01) expressing FLAG-tagged FAS1, the 3×FLAG-URA3 cassette was amplified from pJT1 plasmid^27^ using primers containing 50 bp homology to the FAS1 C terminus (Table S2). The amplified fragment was used to transform the yeast cells by a lithium acetate-based method^25^. Transformants were selected on synthetic media (SD) uracil-dropout plates and confirmed with PCR using a forward primer chosen from within the *FAS1* gene and a reverse primer chosen from within the inserted cassette (Table S2).

To construct *C*. *albicans* yeast strain CaLC5425, the 6xHIS-3xFLAG-HIS cassette was amplified from pLC1085 plasmid^28^ using the primers oLC6912 and oLC6913 which contained 70 bp homology to *C*. *albicans* FAS1 C-terminus (Table S3). The amplicon was then transformed into SN95 (ura3::imm434::URA3/ura3iro1IRO1/iro1his1his1arg4/arg4) strain. Cells were plated on SD + ARG plates and HIS+ transformants were PCR tested for correct integration of 6xHIS-3xFLAG at the C terminus of FAS1 using the primer pairs oLC6914/oLC6915 and oLC6916/oLC6917 (Table S3).

To purify endogenous yeast FAS complexes, 1L of *S*. *cerevisiae* yeast in YPD media were grown in 4L flasks in an Innova 42 shaker (New Brunswick) at 25 °C until OD_660nm_ ~ 2.0, or mid-exponential phase. *C*. *albicans* cells were grown in 600 mL YPD medium in 2 L flasks shaking (Thermo Electron) at 30 °C until an OD_600nm_ ~ 3.0 was reached. Cells were harvested by centrifugation at 4,000 rpm for 10 min, flash frozen in liquid nitrogen and stored at −80C. For purification, cells were resuspended in lysis buffer (200 mM potassium phosphate pH 7.4, 10 mM EDTA, 0.5 mM PMSF, 50 mM β-glycerophosphate, 10 mM NaF, 5 mM aminocaproic acid, 1 mM benzamidine). Lysis was achieved by mechanical disruption at ice-water temperature using a BeadBeater (BIoSpec Products) with bead beating for 30 s and resting for 60 s for a total of 270 s using 0.5 mm diameter glass beads. The lysate was cleared by an initial centrifugation at 4,000 g for 10 min followed by ultracentrifugation at 110,000 g for 30 min. The cleared lysate was filtered using a 0.22 um pore size filter and loaded onto a pre-equilibrated column containing 500 μL of anti-FLAG M2 affinity resin (Sigma-Aldrich). The column was washed 10 times with one column volume of lysis buffer and 10 times with one column volume elution buffer (50 mM Tris-HCl pH 7.4, 150 mM NaCl) and eluted with 3 column volumes of 150 ug/mL 3×FLAG peptide (GenScript) in elution buffer. 1 mM DTT was added to the purified sample after elution. Purity was assessed by Coomassie Blue stained 8–15% gradient SDS-PAGE.

### Activity assays

Activity of purified FAS complexes at 0.2 mg/ml were measured through a spectrophotometric assay monitoring the level of NADPH (Sigma-Aldrich) at 340 nm. The assay mixture contained 100 mM potassium phosphate pH 7.4, 1 mM EDTA, 0.2 mM acetyl-CoA (Sigma-Aldrich), 0.7 mM NADPH, 1 mM DTT in a 100 μl reaction volume. The absorbance at 340 nm was measured for 3 minutes, after which the reaction was initiated by the addition of 30 nmol malonyl-CoA (Sigma-Aldrich) and monitored for 15 minutes at 25 °C. To test the effect of the inhibitor on the enzymatic activity, FAS was incubated with cerulenin (Sigma-Aldrich) at room temperature for 1 h prior to the activity assay. For the positive control reactions (absence of cerulenin), DMSO was added to 1% v/v to match the highest DMSO concentration tested in the inhibitory reactions (*i*.*e*. 100 μM cerulenin). Each curve is normalized against its first point of measurement after addition of malonyl-CoA.

### Electron cryomicroscopy

To prepare samples for imaging via electron microscope, freshly purified FAS complexes were concentrated to 4 mg/mL before applying 3 μl onto the nanofabricated holey sputtered gold grid^29^ with a hole size of ~2 μm. Grid freezing was done in Vitrobot Mark IV (FEI) with 3 sec blotting at 4 °C, 100% humidity and using liquid ethane at liquid nitrogen temperature. For KS-inhibited state, purified FAS at 0.5 mg/ml was incubated with 25 μM of cerulenin for 1 h at room temperature. The sample was then concentrated to 10 mg/ml followed by addition of acetyl-CoA, malonyl-CoA, and NADPH pH-adjusted mixture. The final concentrations were 4 mg/ml FAS, 1 mM acetyl-CoA, 3 mM NADPH, and 1.4 mM malonyl-CoA. Freezing was done as with the Apo sample.

Cryo-grids were screened for ice-thickness, particle distribution, and sample behaviour with a FEI Tecnai F20 field emission electron microscope equipped with a Gatan K2 summit direct detector device (DDD) camera. Images were acquired in counting mode with 1.45 Å/pixel, 2 frames/s for 15 s, and an exposure rate of 1.2 e^−^/Å^2^/frame. Data collection was done with Titan Krios G3 electron microscope. See Table S1 for details on data collection from Titan Krios electron microscope.

### Image processing and model building

Image processing and statistics can be found in Table S1. All motion corrections were done with Alignframe_lmbfgs and Alignpart_lmbfgs^30^ that are implemented in cryoSPARC V2^31^. Particle picking, 2D classification and refinement were also done with cryoSPARC V2. For generating ACP densities inside the reaction chamber, a full FAS complex was modeled using PDB: 2UV8^2^ followed by deletion of all ACP atoms. The modified model was converted to a cryoEM density map using Chimera^32^ and low-pass filtered to 30 Å. *Ab initio* ACP densities were generated using this modified ACP-less map as the initial model with D3 symmetry imposed. All *ab initio* maps were set to a maximum resolution of 12 Å (resolution range 30 to 12 Å, in cryoSPARC V1). These maps are shown and referred to in Fig. 2A,B of the main text. All subsequent high-resolution refinements were done with the corresponding maps from *ab initio* reconstructions with ACP-less initial FAS models and D3 symmetry applied. These high-resolution refinements were done with cryoSPARC V2 nonuniform (NU-) refinement algorithm and the filtered maps were used for model building. In all NU-refinements, the dynamic mask threshold was set to 0.05 (range 0–1, default = 0.2) for better masking and refinement of the ACP densities. The Fourier shell correlation (FSC) curves were calculated using the independently refined half maps with resolution assessed at 0.143 after correcting for the effects of masking maps. Where otherwise stated high-resolution filtered maps (from NU-refinement) are shown in the figures. Scaling and thresholding of different maps were done in Chimera^32^ using ‘vop’ command and volume viewer tool. Model building was done using the high-resolution locally-filtered nonuniform maps and PDB 2UV8^2^ as the initial model for *S*. *cerevisiae* FAS. For *C*. *albicans* FAS, a homology model was built with SWISS-MODEL^33^ using PDB 2UV8^2^ as template and subsequently refined. In both FAS systems, the quality of the ACP density did not allow for confident building of complete atomic models for this mobile domain. Therefore, polyalanine models of ACP were generated. Refinement was done with Phenix^34,35^ real space refinement tool and manual model building was done with Coot^36^. Validation statistics were generated using Molprobity^37^ and EMringer^38^ software implemented within Phenix. All visualizations are done with Chimera^32^ and PyMol^39^. Surface electrostatic potentials for the KS and ER-domains were estimated using the refined atomic models and Coulombic surface coloring tool in Chimera. For surface electrostatics of ACP, homology models were made for both fungal species with SWISS MODEL^33^ using PDB: 2UV8 as template and used for Coulombic surface coloring.

## Supplementary information

Supplementary Tables and Figures

## Data Availability

All cryoEM maps and models have been deposited to PDB with the following codes: *S*. *cerevisiae* Apo: 6U5T, *S*. *cerevisiae* KS-stalled: 6U5U, *C*. *albicans* Apo: 6U5V, *C*. *albicans* KS-stalled: 6U5W.

## References

[1] Lomakin IB, Xiong Y, Steitz TA (2007). The crystal structure of yeast fatty acid synthase, a cellular machine with eight active sites working together. Cell.

[2] Leibundgut M, Jenni S, Frick C, Ban N (2007). Structural basis for substrate delivery by acyl carrier protein in the yeast fatty acid synthase. Science.

[3] Maier T, Leibundgut M, Boehringer D, Ban N (2010). Structure and function of eukaryotic fatty acid synthases. Q. Rev. Biophys..

[4] Fichtlscherer F, Wellein C, Mittag M, Schweizer E (2000). A novel function of yeast fatty acid synthase. Subunit alpha is capable of self-pantetheinylation. Eur. J. Biochem..

[5] Elad N (2018). Structure of Type-I Mycobacterium tuberculosis fatty acid synthase at 3.3 Å resolution. Nat. Commun..

[6] Gipson P (2010). Direct structural insight into the substrate-shuttling mechanism of yeast fatty acid synthase by electron cryomicroscopy. Proc. Natl. Acad. Sci. USA.

[7] Kastritis PL (2017). Capturing protein communities by structural proteomics in a thermophilic eukaryote. Mol. Syst. Biol..

[8] Anselmi C, Grininger M, Gipson P, Faraldo-Gómez JD (2010). Mechanism of Substrate Shuttling by the Acyl-Carrier Protein within the Fatty Acid Mega-Synthase. J. Am. Chem. Soc..

[9] Ye Z, Williams GJ (2014). Mapping a Ketosynthase:Acyl Carrier Protein Binding Interface via Unnatural Amino Acid-Mediated Photo-Cross-Linking. Biochemistry.

[10] Tallorin L (2016). Trapping of the Enoyl-Acyl Carrier Protein Reductase-Acyl Carrier Protein Interaction. J. Am. Chem. Soc..

[11] Nguyen C (2014). Trapping the dynamic acyl carrier protein in fatty acid biosynthesis. Nature.

[12] Konno S, La Clair JJ, Burkart MD (2018). Trapping the Complex Molecular Machinery of Polyketide and Fatty Acid Synthases with Tunable Silylcyanohydrin Crosslinkers. Angew. Chemie Int. Ed..

[13] Johansson P (2009). Multimeric Options for the Auto-Activation of the Saccharomyces cerevisiae FAS Type I Megasynthase. Structure.

[14] Johansson P (2008). Inhibition of the fungal fatty acid synthase type I multienzyme complex. Proc. Natl. Acad. Sci. USA.

[15] Maier T, Leibundgut M, Ban N (2008). The Crystal Structure of a Mammalian Fatty Acid Synthase. Science (80-.)..

[16] Jenni S (2007). Structure of fungal fatty acid synthase and implications for iterative substrate shuttling. Science.

[17] Vernon CN, Hsu RY (1986). The presence of a histidine residue at or near the NADPH binding site of enoyl reductase domain on the multifunctional fatty acid synthetase of chicken liver. Biochim. Biophys. Acta.

[18] Mukherjee S, Katiyar SS (1997). Evidence for the essential histidine at the NADPH binding site of enoyl-CoA reductase domain of pigeon liver fatty acid synthetase. J. Enzyme Inhib..

[19] Nguyen LN, Trofa D, Nosanchuk JD (2009). Fatty acid synthase impacts the pathobiology of Candida parapsilosis *in vitro* and during mammalian infection. PLoS One.

[20] Chayakulkeeree M, Rude TH, Toffaletti DL, Perfect JR (2007). Fatty Acid Synthesis Is Essential for Survival of Cryptococcus neoformans and a Potential Fungicidal Target. Antimicrob. Agents Chemother..

[21] Marreddy Ravi K. R., Wu Xiaoqian, Sapkota Madhab, Prior Allan M., Jones Jesse A., Sun Dianqing, Hevener Kirk E., Hurdle Julian G. (2018). The Fatty Acid Synthesis Protein Enoyl-ACP Reductase II (FabK) is a Target for Narrow-Spectrum Antibacterials for Clostridium difficile Infection. ACS Infectious Diseases.

[22] Zhu Z (2017). Expanding the product portfolio of fungal type I fatty acid synthases. Nat. Chem. Biol..

[23] Gajewski J (2017). Engineering fatty acid synthases for directed polyketide production. Nat. Chem. Biol..

[24] Rossini E, Gajewski J, Klaus M, Hummer G, Grininger M (2018). Analysis and engineering of substrate shuttling by the acyl carrier protein (ACP) in fatty acid synthases (FASs). Chem. Commun..

[25] Mazhab-Jafari Mohammad T., Rohou Alexis, Schmidt Carla, Bueler Stephanie A., Benlekbir Samir, Robinson Carol V., Rubinstein John L. (2016). Atomic model for the membrane-embedded VO motor of a eukaryotic V-ATPase. Nature.

[26] Xie JL (2017). The Candida albicans transcription factor Cas5 couples stress responses, drug resistance and cell cycle regulation. Nat. Commun..

[27] Benlekbir S, Bueler SA, Rubinstein JL (2012). Structure of the vacuolar-type ATPase from Saccharomyces cerevisiae at 11-Å resolution. Nat. Struct. Mol. Biol..

[28] Zhang A (2012). The Tlo Proteins Are Stoichiometric Components of Candida albicans Mediator Anchored via the Med3 Subunit. Eukaryot. Cell.

[29] Marr CR, Benlekbir S, Rubinstein JL (2014). Fabrication of carbon films with <500 nm holes for cryo-EM with a direct detector device. J. Struct. Biol..

[30] Rubinstein JL, Brubaker MA (2015). Alignment of cryo-EM movies of individual particles by optimization of image translations. J. Struct. Biol..

[31] Punjani A, Rubinstein JL, Fleet DJ, Brubaker M (2017). A. cryoSPARC: algorithms for rapid unsupervised cryo-EM structure determination. Nat. Methods.

[32] Pettersen EF (2004). UCSF Chimera?A visualization system for exploratory research and analysis. J. Comput. Chem..

[33] Waterhouse A (2018). SWISS-MODEL: homology modelling of protein structures and complexes. Nucleic Acids Res..

[34] Adams PD (2010). *PHENIX*: a comprehensive Python-based system for macromolecular structure solution. *Acta Crystallogr*. Sect. D Biol. Crystallogr..

[35] Afonine PV (2018). Real-space refinement in *PHENIX* for cryo-EM and crystallography. Acta Crystallogr. Sect. D Struct. Biol..

[36] Emsley P, Lohkamp B, Scott WG, Cowtan K (2010). Features and development of *Coot*. *Acta Crystallogr*. Sect. D Biol. Crystallogr..

[37] Chen VB (2010). MolProbity: all-atom structure validation for macromolecular crystallography. Acta Crystallogr. D. Biol. Crystallogr..

[38] Barad BA (2015). EMRinger: side chain-directed model and map validation for 3D cryo-electron microscopy. Nat. Methods.

[39] The PyMOL Molecular Graphics System, Version 2.0 Schrödinger, LLC. at, https://pymol.org/2/support.html? (2019)

