# Supplementary material for: Electron cryomicroscopy observation of acyl carrier protein translocation in type I fungal fatty acid synthase
Source: Sci Rep. 2019 Sep 10;9:12987. doi: 10.1038/s41598-019-49261-3 (PMC6736866; doi:10.1038/s41598-019-49261-3)
Supplement: Supplementary file 1 — Supplementary Tables and Figures [file 41598_2019_49261_MOESM1_ESM.pdf]

Electron cryomicroscopy observation of acyl carrier protein translocation in type I fungal fatty acid synthase

Jennifer W. Lou‡, Kali R. Iyer†, S. M. Naimul Hasan‡, Leah E. Cowen†, Mohammad T. Mazhab-Jafari‡*

‡Department of Medical Biophysics, University of Toronto, Princess Margaret Cancer Research Institute, Toronto, Ontario, Canada. †Department of Molecular Genetics, University of Toronto, Toronto, Ontario, Canada

* Correspondence: Mohammad.Mazhab-Jafari@uhnresearch.ca

Supplementary Information:

Supplementary Table 1……………………………..……..……2

Supplementary Table 2……………………………………………2

Supplementary Table 3…………………………...……………2

Supplementary Figure 1……………………………………….…..3

Supplementary Figure 2……………………………..…...………..5

Supplementary Figure 3……………………………..……………..7

Supplementary Figure 4………………………………….…………..8

Supplementary Figure 5………………………..……….....………..10

Supplementary Figure 6………………………..……………………..11

Supplementary Figure 7………………………..……………………..13

Supplementary Figure 8………………………..……………………..14

Supplementary Table 1) Data collection and statistics.

**Data Collection**	**S. cerevisiae FAS (Apo)**	**S. cerevisiae FAS (KS-stalled)**	**C. albicans FAS (Apo)**	**C. albicans FAS (KS-stalled)**
Microscope	Titan Krios G3	Titan Krios G3	Titan Krios G3	Titan Krios G3
Camera	FEI Falcon 3EC	FEI Falcon 3EC	FEI Falcon 3EC	FEI Falcon 3EC
Voltage	300 kV	300 kV	300 kV	300 kV
Magnification	75,000×	75,000×	75,000×	75,000×
Pixel size	1.06 Å	1.06 Å	1.06 Å	1.06 Å
Exposure	43 electrons/Å^2	43 electrons/Å^2	43 electrons/Å^2	43 electrons/Å^2
Exposure rate	0.8 Electron/Å^2/Sec	0.8 Electron/Å^2/Sec	0.8 Electron/Å^2/Sec	0.8 Electron/Å^2/Sec
Number of Frames	30	30	30	30
Defocus Range	0.6-2.5μm	0.6-2.5μm	0.6-2.5μm	0.6-2.5μm
**Image Processing**				
Frame motion correction	Alignframe_lmbfgs	Alignframe_lmbfgs	Alignframe_lmbfgs	Alignframe_lmbfgs
CTF estimation	CTFFIND4	CTFFIND4	CTFFIND4	CTFFIND4
CTF cutoff	5 Å	5 Å	5 Å	5 Å
Particle picking software	cryoSPARC	cryoSPARC	cryoSPARC	cryoSPARC
Micrographs Used	4,075	4,056	1,123	1,310
Particle image motion correction	Alignpart_lmbfgs	Alignpart_lmbfgs	Alignpart_lmbfgs	Alignpart_lmbfgs
Particles Contributed	637,823	594,818	92,958	24,417
Reconstruction Software	cryoSPARC	cryoSPARC	cryoSPARC	cryoSPARC
Symmetry Applied	D3	D3	D3	D3
Global Resolution (FSC = 0.143)	2.9	2.8	2.8	3.3
**Model Building**				
Modeling Software	Coot, Phenix	Coot, Phenix	Coot, Phenix	Coot, Phenix
Number of Residues build	3,647	3,647	3,376	3,376
RMS (Bond)	0.006	0.008	0.013	0.006
RMS (Angles)	1.13	1.2	1.44	1.17
Ramachandaran Outliers	0.03%	0.03%	0.06%	0.09%
C-beta outliers	0.00%	0.00%	0.00%	0.00%
Rotamer Outliers	0.22%	0.48%	0.93%	0.20%
Clashscore	5.79	5.99	6.2	4.5
MolProbity Score	1.57	1.62	1.8	1.56
EMRinger Score	1.95	2.06	3.17	3.11
PDB ID	XXXX	XXXX	XXXX	XXXX

Supplementary Table 2) Primers for tagging 3' end of *FAS1* gene in *S. cerevisiae*.

Name	Sequence (5' -> 3')
Homologous recombination F	CCGAACCTATCAAGGAAATCATCGACAACTGGGAAAAGTATGAACAATCCGACTACAAAGACCATGACGG
Homologous recombination R	CAGGAGTTTCAAAGTTAAATATTTCTTACGGTTATATAATCACTTAAGAAAATATCATCGATGAATTCGAGCTCG
Confirmation F	GAAGGTTGCTAGATTGGCCG
Confirmation R	GAGCGACCTCATACTATACC

Supplementary Table 3) Primers for tagging 3' end of *FAS1* gene in *C. albicans*.

Name	Sequence (5' -> 3')
oLC6912	CCAATCAGTTTATGATTTGACTAAATCGGAAAAAATCAAGAGTATTTTAGATAACTGGGAACAATACGAAGGTCGACGGATCCCC
oLC6913	ATAACAGGTCCTTTAAATAGCAAGTAAATAGAAATTTTATACATTTATTTATTATCTATACATTCTAAGTTCGATGAATTCGAGCTCG
oLC6914	TGCTGTCATTCCATTGAAGG
oLC6915	TAACTTCTGTCTCCTCATCCTC
oLC6916	TTTAAAGTCAATAGGCATTCTCG
oL6917	AGCCCAACTGGTAAAAAGCA

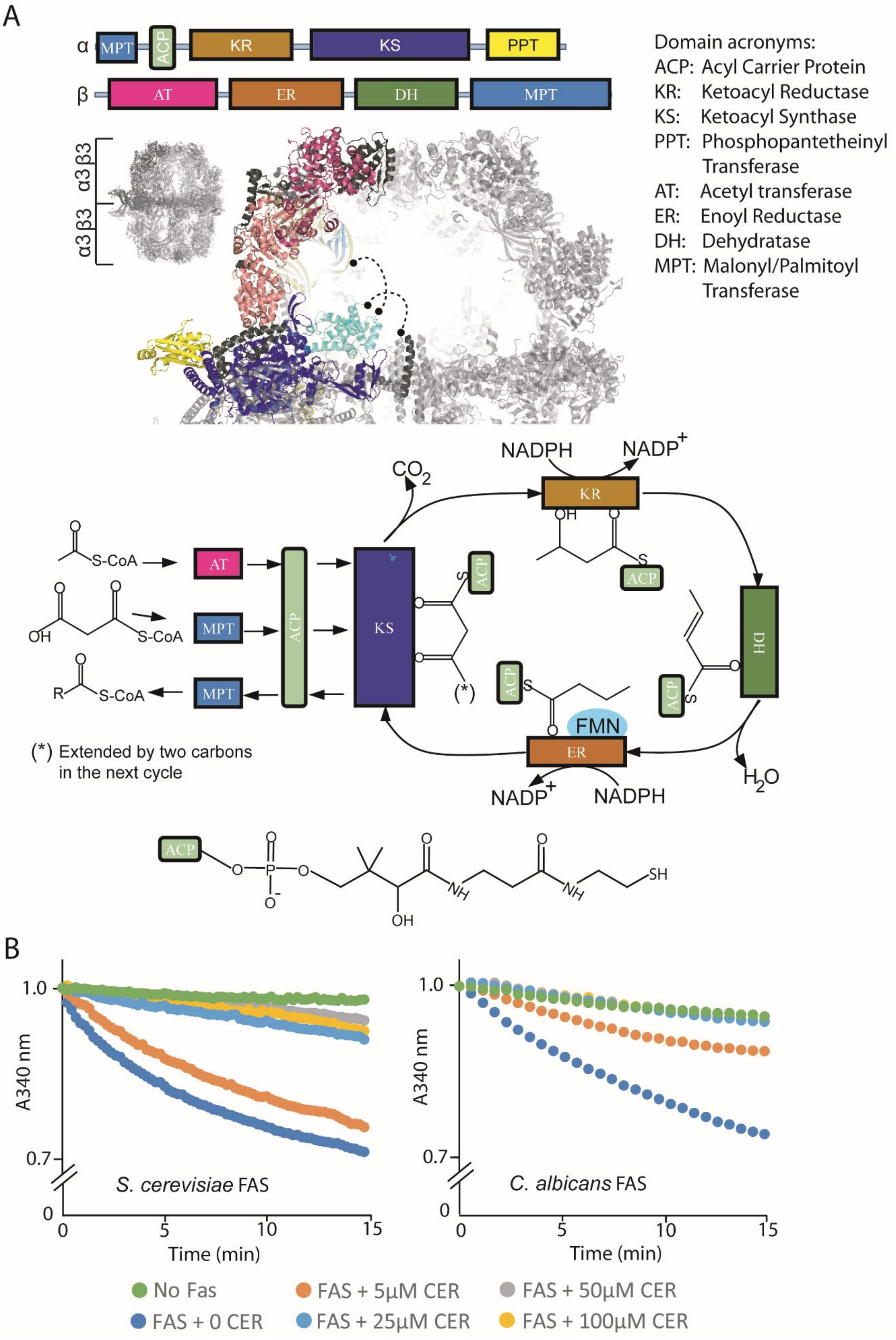
A
α
MPT
ACP
KR
KS
PPT
β
AT
ER
DH
MPT
α3β3
α3β3
Domain acronyms:
ACP: Acyl Carrier Protein
KR: Ketoacyl Reductase
KS: Ketoacyl Synthase
PPT: Phosphopantetheinyl Transferase
AT: Acetyl transferase
ER: Enoyl Reductase
DH: Dehydratase
MPT: Malonyl/Palmitoyl Transferase
NADPH
NADP+
CO2
KR
OH
S
ACP
S-CoA
AT
OH
S-CoA
MPT
ACP
KS
DH
R
S-CoA
MPT
(*)
FMN
ER
H2O
NADP+
NADPH
(*) Extended by two carbons in the next cycle
ACP
O
P
O
OH
NH
NH
SH
B
1.0
0.7
0
A340 nm
0
5
10
15
Time (min)
S. cerevisiae FAS
C. albicans FAS
No Fas
FAS + 0 CER
FAS + 5μM CER
FAS + 25μM CER
FAS + 50μM CER
FAS + 100μM CER

Supplementary Figure 1. (A) Top panel: schematics of the domain organization of *S. cerevisiae* and *C. albicans* FAS. Middle panel: atomic model of the reaction chamber with ACP bound proximal to the KS-domain (PDB: 2PFF[17]). Same color coding as the domain schematics. Bottom panel: schematics of the reaction cycle for palmitoyl-CoA biosynthesis is shown (adopted with modifications from[18]) and Chemical structure of the phosphopantetheine arm is shown at the bottom (drawn with ChemSketch, Advanced Chemistry Development, Inc.) (B) Sensitivity of purified *S. cerevisiae* (left) and *C. albicans* (right) FAS to antibiotic cerulenin. The reactions were done in triplicates and a representative activity curve is shown for each condition tested.

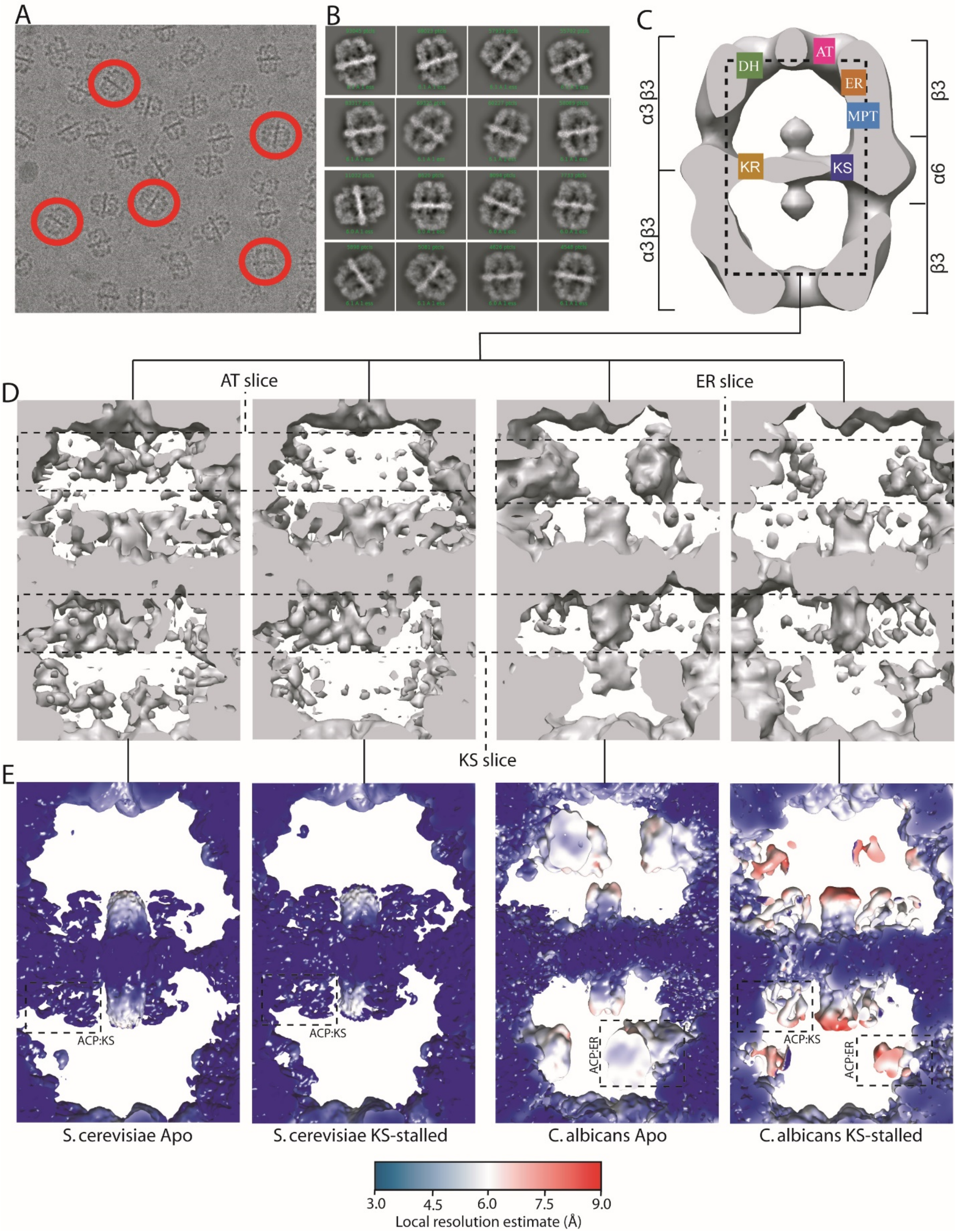

Supplementary Figure 2. (A) Example of an aligned and averaged micrograph of *S. cerevisiae* FAS particles in vitreous ice. (B) From top to bottom row, 2D classes of *S. cerevisiae* Apo, KS-stalled, and

C. albicans Apo and KS-stalled states. (C-E) Scheme for generating *ab initio* ACP densities inside the reaction chamber. Maps are sliced through the long axis (dashed box) to show the interior of the reaction chambers. D3 symmetry applied. (C) initial ACP-less model, (D) *ab initio* reconstructions with experimental particle images. Maps are scaled for each fungal species and shown at identical threshold. Slices through the reaction chamber for ACP at AT, ER, and KS catalytic sites are highlighted with dashed box. (E) high resolution refined maps colored based on local resolution estimate. Representative ACP domains are highlighted with dashed boxes with the name of the interacting catalytic site shown as ACP:catalytic center (*e.g.* ACP:KS).

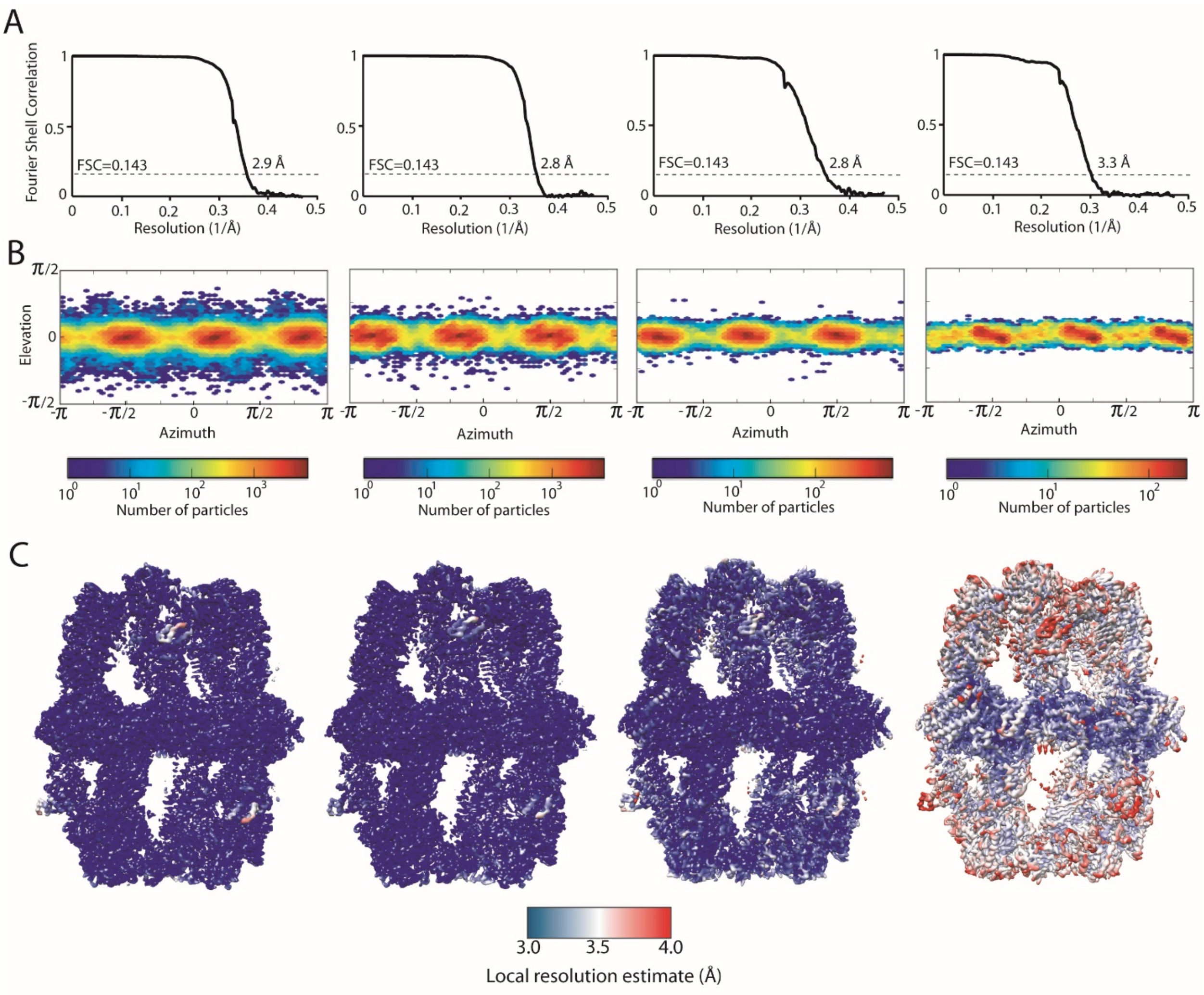

Supplementary Figure 3. Quality of the high-resolution maps used in model building. In all panels, left to right: *S. cerevisiae* Apo and KS-stalled, and *C. albicans* Apo and KS-stalled maps. (A) mask-corrected FSC curves, (B) orientation distributions of particle images, and (C) local resolution estimates of the final high-resolution refined maps used in model building. These maps are the same as shown in slices in supplementary Figure 2E but with coloring for different resolution range as indicated.

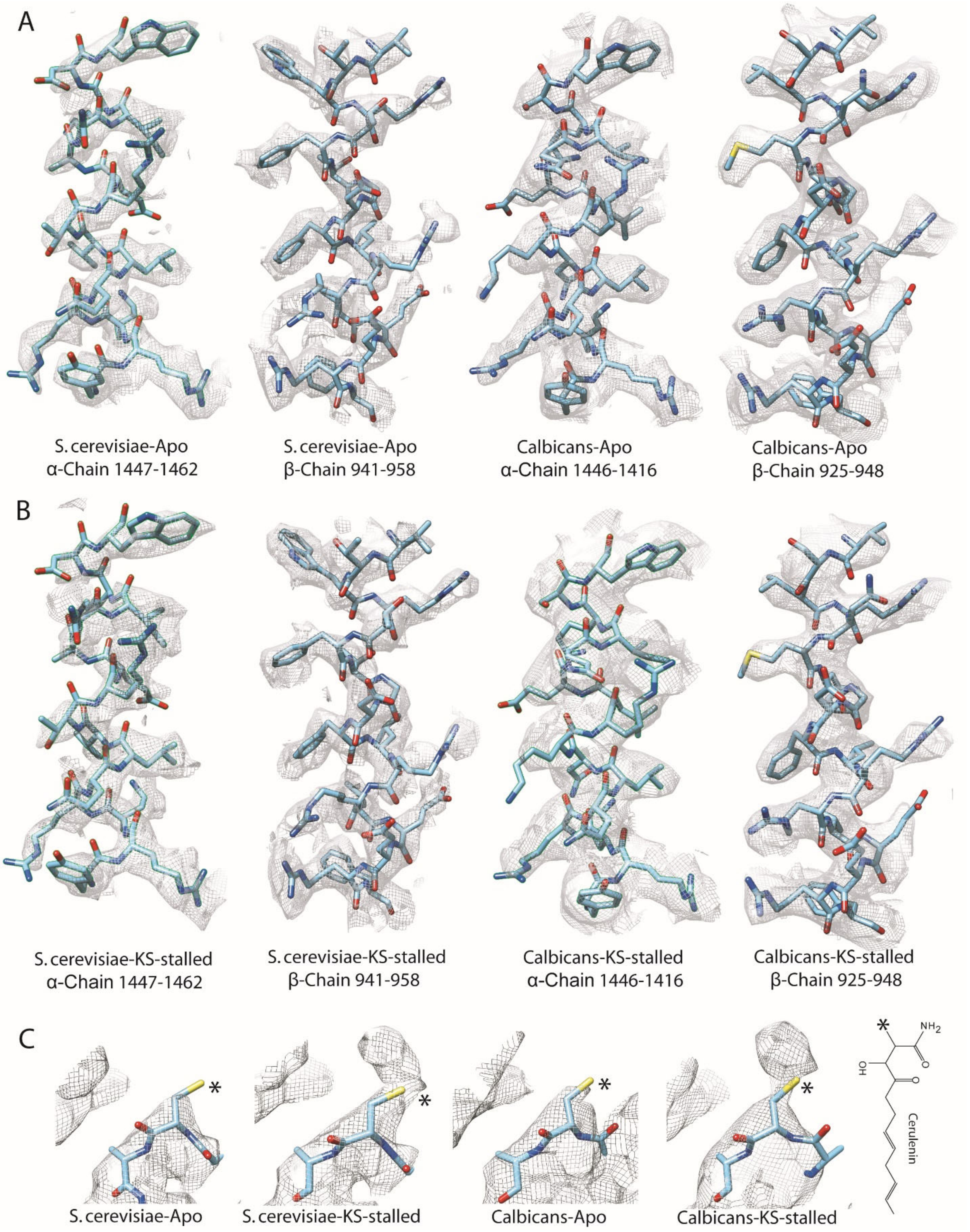

Supplementary Figure 4. Quality of model to map fit. Examples of atomic model fit to high resolution refined maps are shown for both α- β-chains as indicated with respective residue range for (A) Apo and (B) KS-stalled state of each fungal species. (C) appearance of extra density on -SH

functional group (*) of catalytic cysteine of the KS-domain upon inhibition with cerulenin (chemical structure shown to the far right, drawn with ChemSketch, Advanced Chemistry Development, Inc.) for each fungal species. Densities within 5 Å for the catalytic cysteine and the two flanking residues are shown.

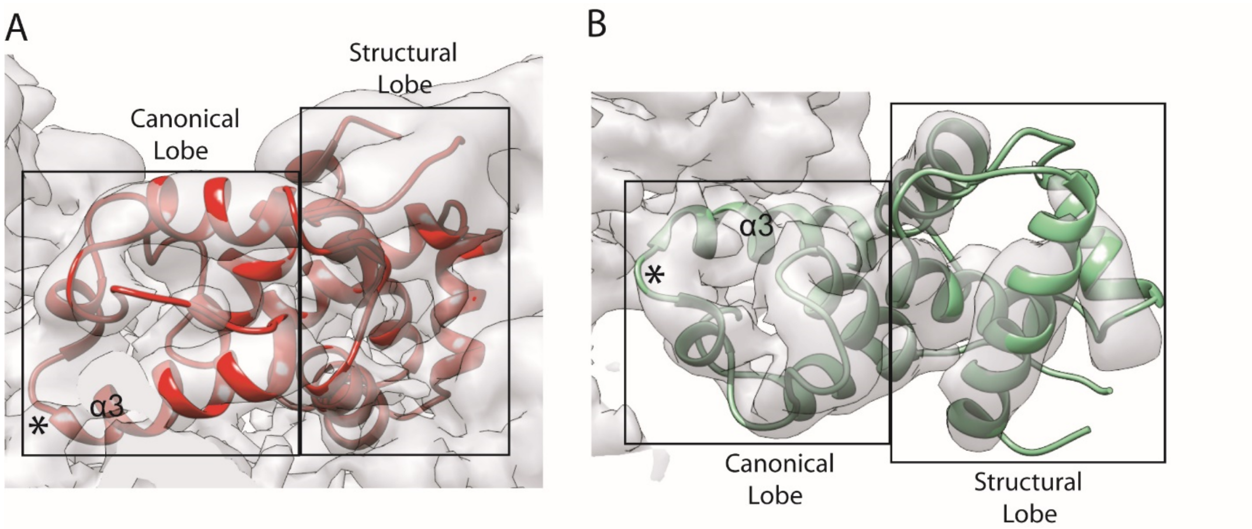

Supplementary Figure 5. Fitting of the ACP domains to their corresponding densities near (A) KS- and (B) ER- domains in *S. cerevisiae* and *C. albicans* FAS, respectively. * represents the position of the phosphopantetheine arm.

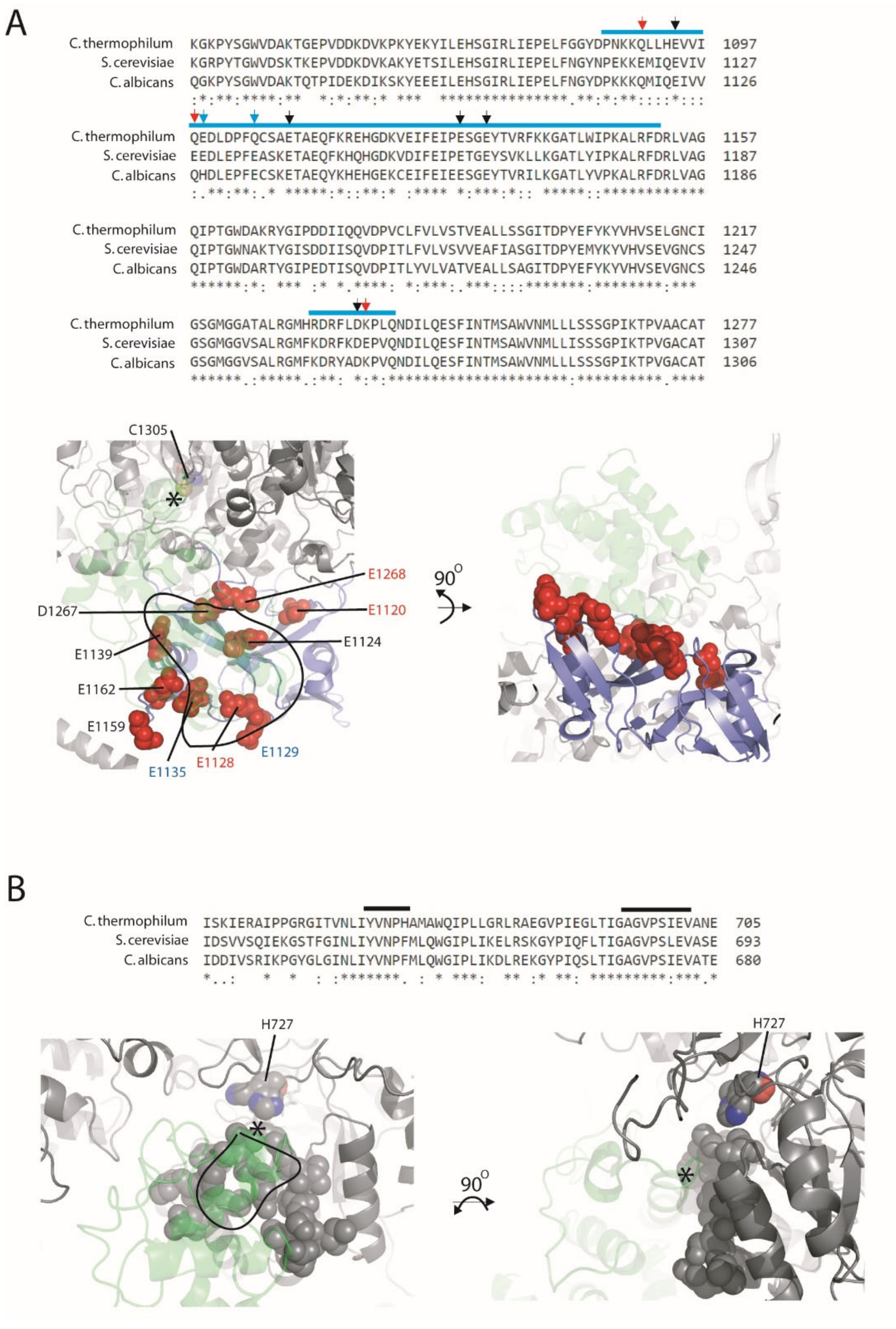

Supplementary Figure 6. (A) Negative charged residues in the ACP binding site of the KS-domain for the structural lobe of ACP. Sequence alignment is shown for the three fungal species. The alignment is done on full-length α-chains of each species using Clustal Omega[19] and only the indicated portion is shown. The KS region involved in interaction with ACP structural lobe is highlighted with cyan on top of the sequences and in the structure (bottom panel, model *S. cerevisiae* Apo). ACP is shown as transparent green. Black, blue, and red arrowheads represent KS acidic residues (facing ACP structural lobe) that are present in the three fungal species, only in two (including *S. cerevisiae*), and only in *S. cerevisiae*, respectively. These residues are highlighted on the structure with the same

colored residue names (B) ER residues lining the ACP binding sites for the canonical lobe of ACP are highlighted with black bars in the sequence alignment for the three fungal species and shown with sphere in the model of *C. albicans* in the Apo state. Sequence alignment done as above but for the β-chain. ACP is shown as transparent green. * represents the position of the phosphopantetheine arm.

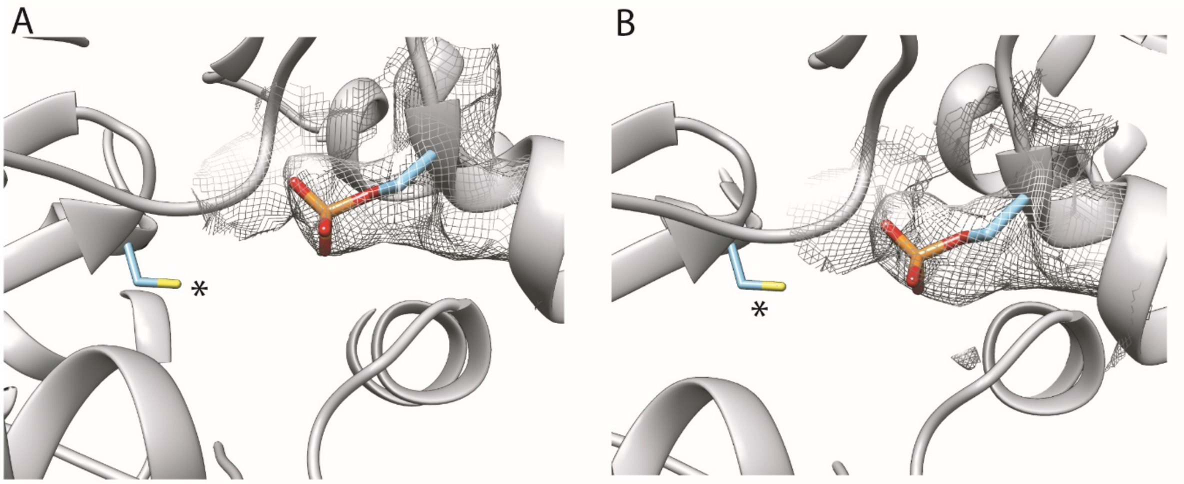

Supplementary Figure 7. Density of the phosphopantetheine arm, in the vicinity of the KS-catalytic cavity. cryoEM density maps in (A) Apo and (B) KS-stalled states of *S. cerevisiae* FAS are shown within 5 Å of the partial model of the phosphopantetheine arm at different thresholds to highlight model-to-map fit. * represents the catalytic cysteine of the KS domain.

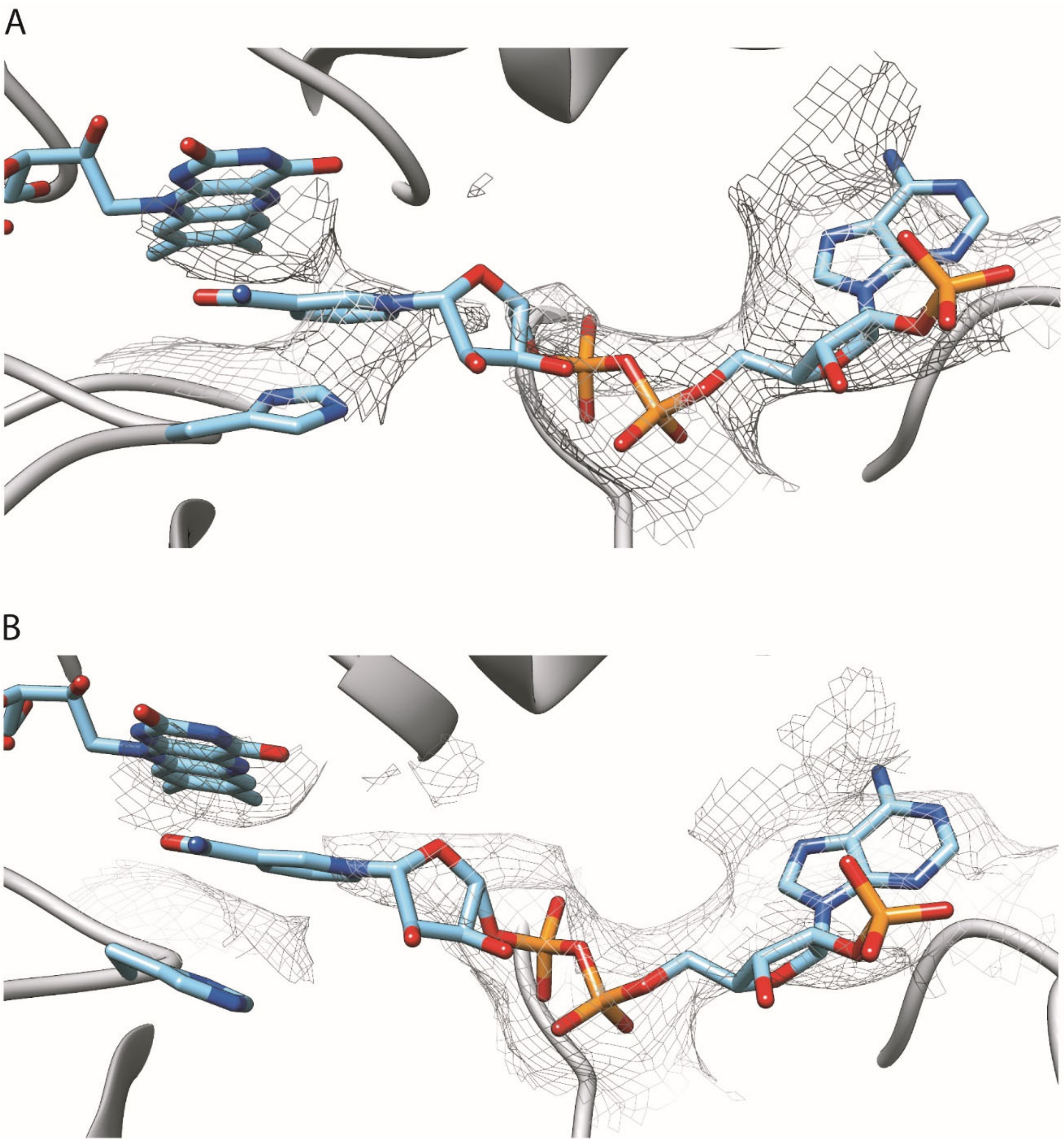

Supplementary Figure 8. cryoEM densities of the NADPH molecule in the ER catalytic sites in the KS-stalled states of (A) *C. albicans* and (B) *S. cerevisiae*.
